# Supplementary material for: Coating Methods of Carbon Nonwovens with Cross-Linked Hyaluronic Acid and Its Conjugates with BMP Fragments
Source: Polymers (Basel). 2023 Mar 21;15(6):1551. doi: 10.3390/polym15061551 (PMC10054264; doi:10.3390/polym15061551)
Supplement: Supplementary file 1 [file polymers-15-01551-s001.zip › polymers-2218281-supplementary.pdf]

Coating methods of carbon nonwovens with cross-linked hyaluronic acid and its conjugates with BMP-fragments

Sylwia Magdziarz [1],*, Maciej Boguń [2] and Justyna Frączyk [1]

[1] Institute of Organic Chemistry, Faculty of Chemistry, Lodz University of Technology, Zeromskiego 116, 90-924 Lodz, Poland

[2] Łukasiewicz – Lodz Institute of Technology, Sklodowskiej-Curie 19/27, 90-570 Lodz, Poland

*Correspondence: sylwia.magdziarz@p.lodz.pl;

Supplementary Information

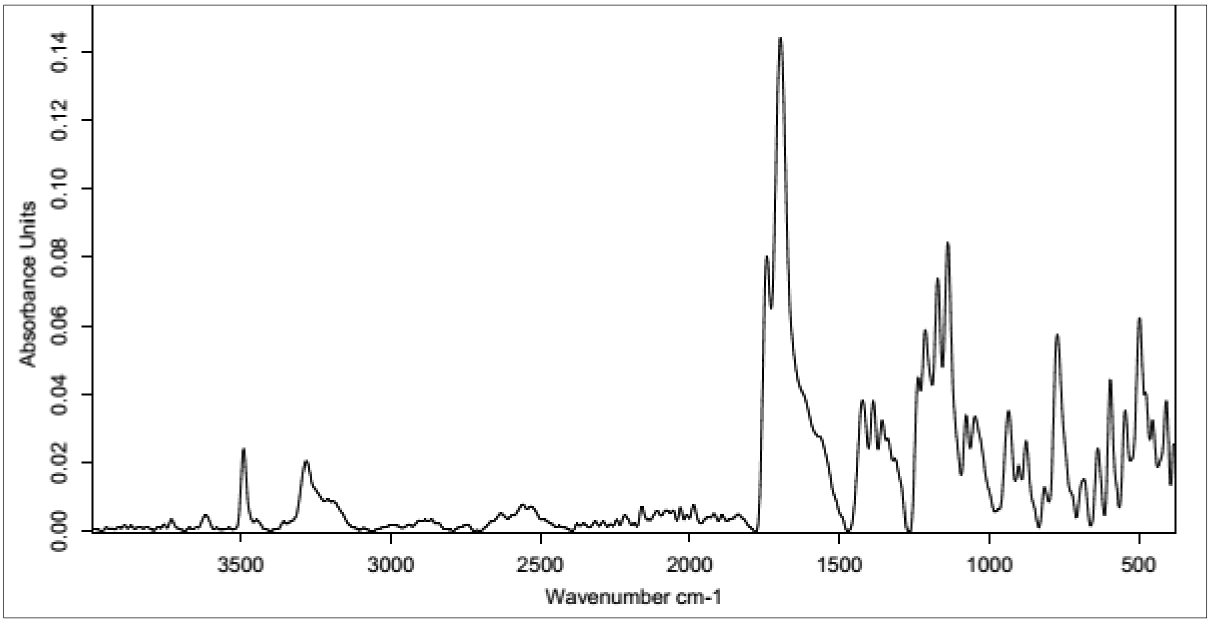

Figure S1. IR spectrum of carbon nonwoven coated with 1% hyaluronic acid and cross-linked with 5% citric acid.

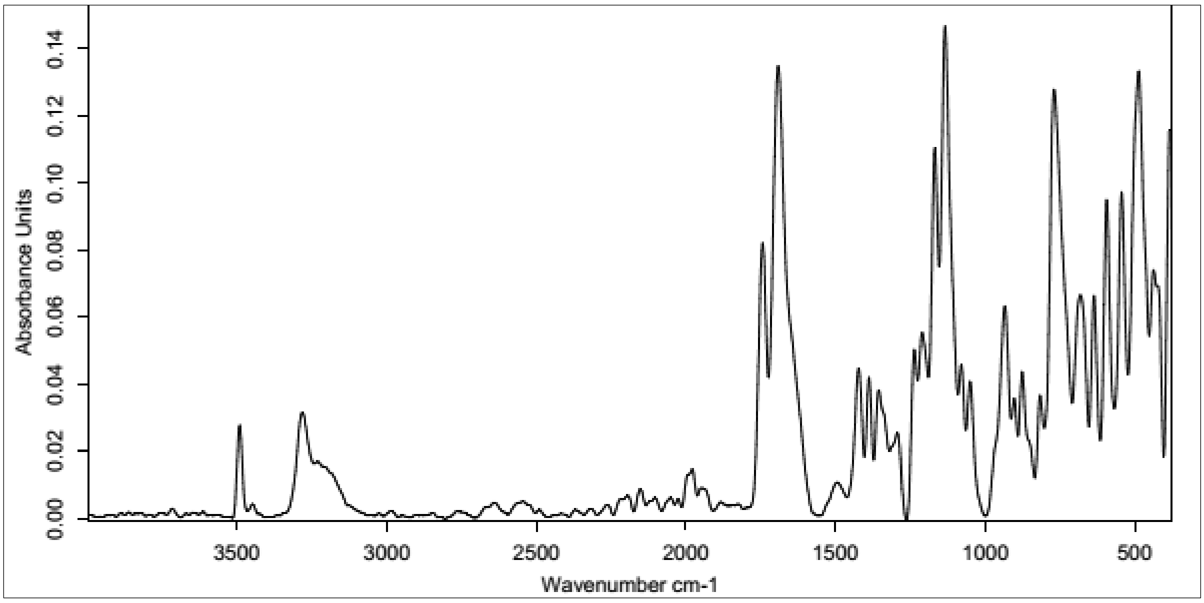

Figure S2. IR spectrum of carbon nonwoven coated with 1% hyaluronic acid and cross-linked with 20% citric acid.

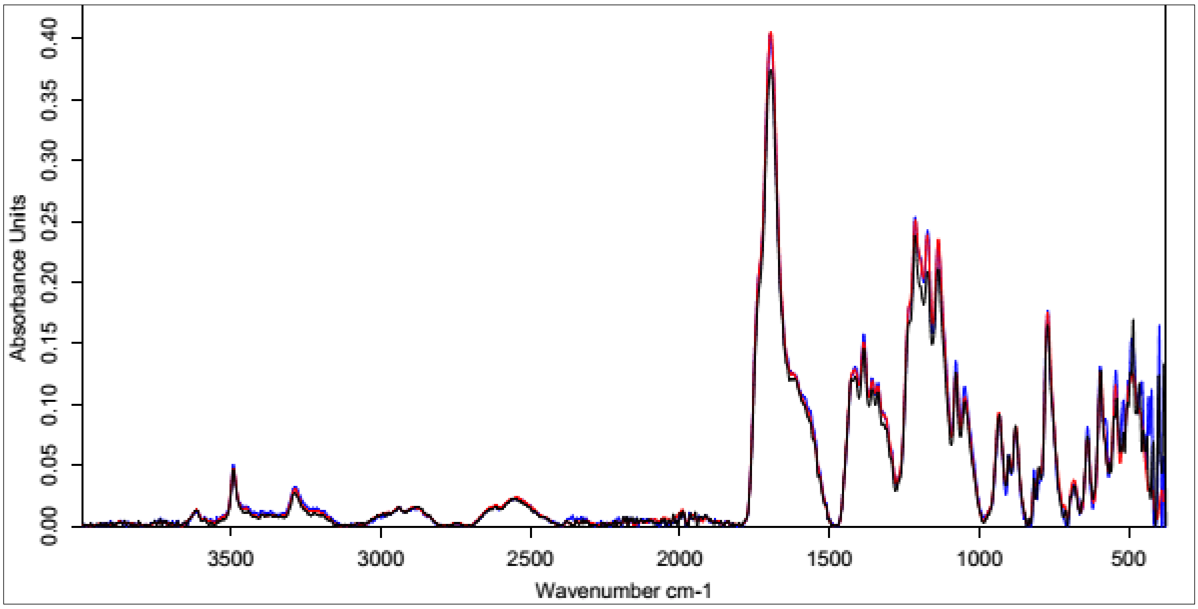

Figure S3. IR spectrum of carbon nonwoven coated with hyaluronic acid of different concentrations: 0,5% (black line), 1% (red line), 2% (blue line) and cross-linked with BDDE.

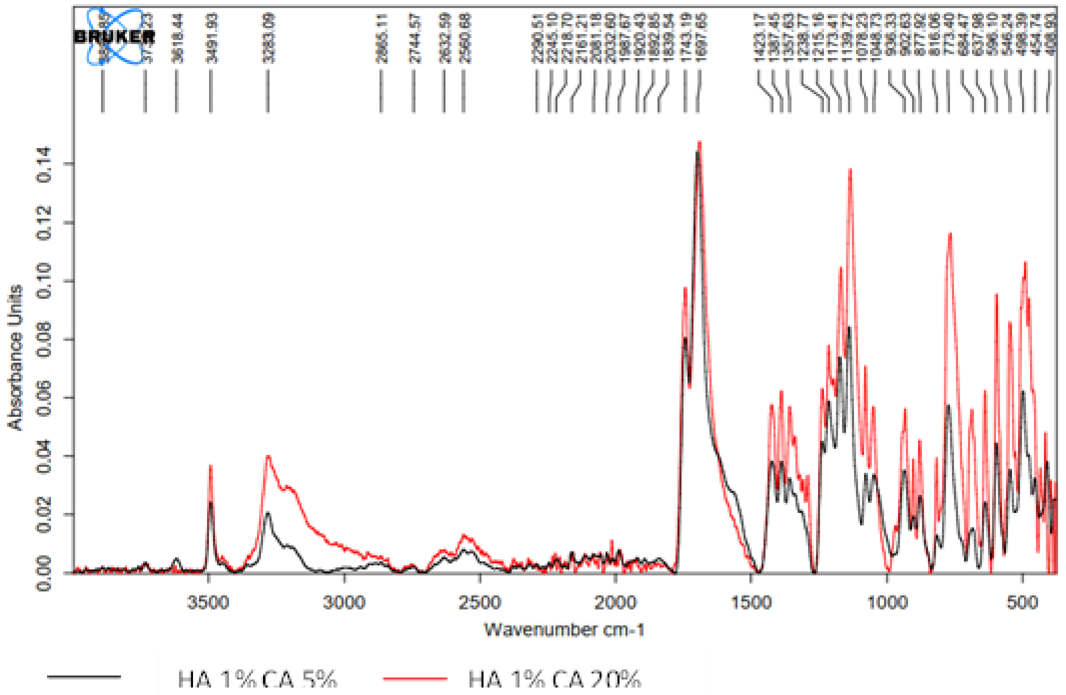

Figure S4. IR spectrum for 1% hyaluronic acid-coated carbon nonwoven samples cross-linked with citric acid of different concentrations.

Synthesis of H-KRMVRISRSL-OH

General Procedure

The peptide was synthesized following a standard solid phase procedure using the Fmoc/tBu protecting group strategy.

Incorporation of the C-Terminal Amino Acid into the 2′-chloro-chlorotrityl Resin

The synthesis was carried out using 0.5 g of the resin. 2′-Chloro-chlorotrityl resin was swelled in DCM for 1 h. The Fmoc-Leu-OH (1.5 fold molar excess with respect to the resin with a 1 mmol/g loading) was dissolved in DCM (10 mL per 1 g of resin) and DIPEA (3 fold molar excess with respect to the amount of resin) was added. The solution was added to the resin and shaken for 1 h. Then, the resin was filtered off and washed with DCM (3 × 3 mL), a mixture of DCM, MeOH and DIPEA 17:2:1 by volume (10 mL), DMF (3 × 3 mL) and DCM (3 × 3 mL). Same procedure has been used in case of the other polypeptide synthesis – The Fmoc-Pro-OH has been added as the *C*-terminal amino acid.

Determination of the Loading of the 2′-chloro-chlorotrityl Resin

An aliquot (6.11 mg) of the resin was placed in a 10 mL volumetric flask and DCM (0.4 mL) and piperidine (0.4 mL) were added. The suspension was left for 30 min. Then MeOH (1.6 mL) was added and the DCM was made up to the mark. The absorbance has been measured in a range 200–400 nm. As a reference sample, a solution prepared in a manner analogous to the proper sample was used. Based on the absorbance value at 301 nm, the resin density was determined based on the formula:

$$Obs\left[\frac{mmol}{g}\right] = \frac{A_{301}}{0.78 * m_{resin}\,[mg]} \quad \text{(S1)}$$

The loading of the resin with Fmoc-Leu-OH was 0.4 mmol/g.
The loading of the resin with Fmoc-Pro-OH was 0.4 mmol/g.

Removal of the Fmoc Protecting Group

The Fmoc group was removed using a 25% piperidine solution. The resin was treated with a 25% solution of piperidine in DMF (5 mL per 1 g of resin). The resin was shaken for 15–20 min. The progress of the reaction was monitored by the Kaiser test.

Incorporation of Further Amino Acids Using DMT/NMM/TosO⁻ as a Coupling Reagent

The appropriate blocked amino acid was weighed out with a threefold molar excess to the resin loading. Then DMT/NMM/$TosO^-$ was weighed out also using a 3 times molar excess to the resin loading. The amino acid and reagent were dissolved in DMF (approx. 10 mL per 1 g of resin). A measured volume of NMM was added to the solution using a sixfold molar excess to the resin loading. After the reagents had dissolved, the solution was added to the resin (with a free amino group) and shaken depending on the amino acid for 1–4 h. The progress of the reaction was monitored by the Kaiser test (the attachment of the amino acid is evidenced by the lack of navy blue color of the resin grains). When incomplete conversion was found, the coupling reaction was repeated. The solution was then removed and the resin was washed sequentially with DMF (3 × 3 mL) and DCM (3 × 3 mL).

Cleavage of the Peptide from the Resin and Product Isolation

After the peptide was synthesized and the Fmoc group removed from the N-terminal amino acid, the thoroughly dried resin was transferred to a flask and the cleavage mixture (approx. 10 mL per 1 g resin) was added. A cleavage mixture of TFA/H_2O/TIS in a volume ratio of 95/2.5/2.5 was used. The suspension was intensively stirred on a magnetic stirrer for 4 h. The polymer was then filtered off the peptide solution. The solution was evaporated under a stream of nitrogen gas. Diethyl ether (10 mL) was added to the residue. The precipitated crude product was centrifuged and decanted. The product was then dissolved in water and freeze-dried.

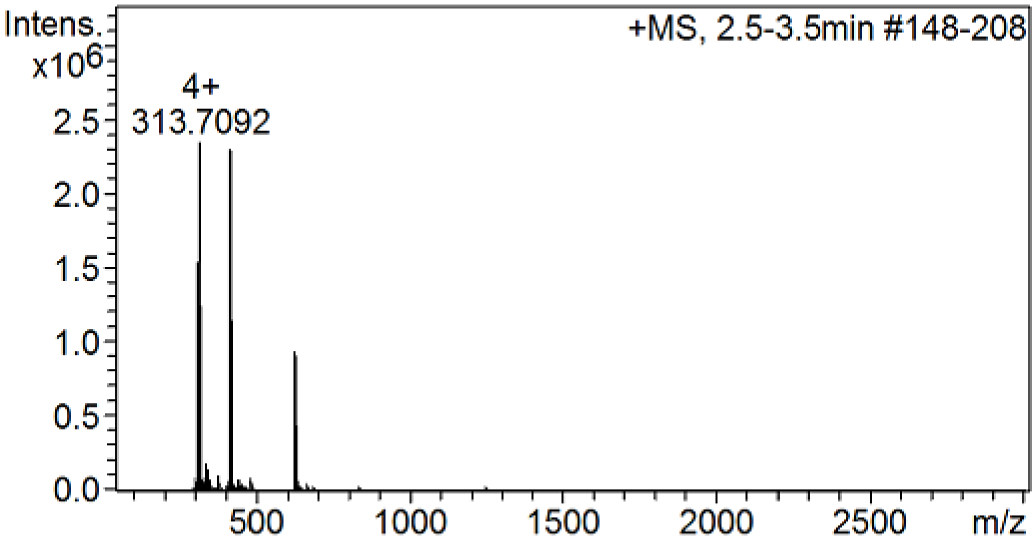

Figure S5. MS spectrum of H-KRMVRISRSL-OH
